# De-Implementing Opioid Use and Implementing Optimal Pain Management Following Dental Extractions (DIODE): Protocol for a Cluster Randomized Trial

**DOI:** 10.2196/24342

**Published:** 2021-04-12

**Authors:** D Brad Rindal, Stephen E Asche, Jan Gryczynski, Sheryl M Kane, Anjali R Truitt, Tracy L Shea, Jeanette Y Ziegenfuss, Robert P Schwartz, Donald C Worley, Shannon G Mitchell

**Affiliations:** 1 HealthPartners Institute Bloomington, MN United States; 2 Friends Research Institute Inc Baltimore, MD United States

**Keywords:** analgesics, opioid, prescriptions, tooth extraction, pain, postoperative, dentistry, oral surgery, shared decision-making, health communications, implementation science

## Abstract

**Background:**

Overdose deaths from prescription opioid analgesics are a continuing crisis in the United States. Opioid analgesics are among the most frequently prescribed drugs by dentists. An estimated 5 million people undergo third-molar extractions in the United States each year, resulting in postoperative pain. Studies show that, in most cases, the combination of ibuprofen and acetaminophen is an effective alternative to commonly prescribed opioid analgesics for the management of postextraction pain. Nevertheless, many dentists routinely prescribe opioids after dental extractions.

**Objective:**

We describe the rationale, design, and methods for a randomized trial of interventions designed to de-implement opioid prescribing by dentists while implementing effective nonopioid analgesics following dental extractions.

**Methods:**

Using a prospective, 3-arm, cluster randomized trial design with dentists as the unit randomized and patient-level prescribing data as the primary outcome, we will compare different strategies to reduce the reliance on opioids and increase the use of alternative pain management approaches utilizing information support tools aimed at both providers and their patients. The study will test the efficacy of 2 interventions to decrease opioid prescribing following dental extractions: clinical decision support with (CDS-E) and without patient education (CDS). Providers will be randomized to CDS, CDS-E, or standard practice. Patient-level outcomes will be determined via review of comprehensive electronic health records. We will compare study arms on differential change in prescribing patterns from pre- to postimplementation of the intervention. The primary outcome of interest is a binary indicator of whether or not the patient received an opioid prescription on the day of the extraction encounter. We will also examine recommendations or prescriptions for nonopioid analgesics, patients’ perceptions of shared decision making, and patients’ pain experiences following the extraction.

**Results:**

The HealthPartners Institutional Review Board approved the study. All study materials including the CDS and patient education materials have been developed and pilot tested, and the protocol has been approved by the National Institute of Dental and Craniofacial Research. The intervention was implemented in February 2020, with 51 dentists who were randomized to 1 of the 3 arms.

**Conclusions:**

If the intervention strategies are shown to be effective, they could be implemented more broadly in dental settings with high levels of opioid prescribing.

**Trial Registration:**

ClinicalTrials.gov NCT03584789, https://clinicaltrials.gov/ct2/show/NCT03584789

**International Registered Report Identifier (IRRID):**

DERR1-10.2196/24342

## Introduction

The United States is experiencing an epidemic of prescription drug overdose deaths, with deaths associated with prescription pain relievers of particular concern [1]. Drug overdose has become a leading cause of accidental death in the United States [2]. Between 2000 and 2015, the rate of deaths from drug overdoses increased 137%, including a 200% increase in the rate of overdose deaths involving opioids (opioid pain relievers and heroin) [3]. Unnecessary opioid prescribing is one of the factors driving this epidemic.

The Centers for Disease Control and Prevention strongly recommends fundamental changes in prescribing practices in order to address this public health emergency [4]. Opioid analgesics are among the most frequently prescribed drugs by dentists [5]. For example, an estimated 5 million people undergo third-molar extractions in the United States each year, resulting in postoperative pain, swelling, and discomfort, even when surgical complications are not present [6]. As part of postoperative dental pain management following third-molar (ie, wisdom teeth) surgery, dentists often introduce patients under 25 years of age to prescription opioids for the first time, which can lead to longer-term substance use [7]. In addition, when opioids go unused by the patient, they are sometimes taken by family members or friends, which can also lead to misuse or abuse [8].

Comprehensive reviews of research evidence have concluded that nonsteroidal anti-inflammatory drugs (NSAIDs) are remarkably effective analgesics for relieving postoperative dental pain and that opioid analgesics have a high incidence of adverse effects [9-11]. Evidence concludes that the combination of the NSAID ibuprofen and acetaminophen (APAP) provides analgesia that is at least equivalent to that of commonly prescribed opioid combination formulations [12]. Thus, NSAID+APAP provides a viable and evidence-based pain management alternative to prescription opioids [12]. Nevertheless, most dentists report that they prescribe opioid medications such as hydrocodone or oxycodone following third-molar extractions [13,14]. Many dentists appear to underestimate the immediate risks and the long-term harms associated with prescription opioids, even as they overestimate opioids’ therapeutic benefits [15]. Strategies to support dentists in de-implementing their overreliance on prescription opioids following dental extractions in favor of safer alternatives are urgently needed.

Various strategies are being examined to reduce reliance on and misuse of prescribed opioids for management of pain following medical procedures [16,17]. Self-management skills such as patient education, decision making, and forming a patient-provider partnership have long been associated with chronic disease management [18] but can be effective components of acute pain management as well. While patient-provider shared decision making is considered the preferential choice when no clear treatment option is optimal [19], involving the patient in the decision process has distinct advantages with respect to medication adherence as well [20]. Collaborative decision making involving the patient and the dentist holds the potential to decrease reliance on opioid medications in favor of recommended alternatives [21]. The study team will utilize this strategy to engage patients in the decision-making process related to the choice of analgesics to manage their pain following dental extractions.

A well-designed clinical decision support (CDS) system can support dentists in providing optimal pain management for patients without resorting to opioids when a safer alternative would suffice. CDS provides pertinent clinical information to the dentist. It can provide evidence-based information and guidance in the form of prompts and reminders to inform clinical decisions about prescriptions. CDS also provides the advantage of ensuring fidelity of the implementation strategy.

The CDS incorporates novel design features that will save significant time, ensure good fit into the dental care workflow, and facilitate the delivery of personalized care by providing the patient’s relevant medical history, informing treatment decisions, and promoting more evidence-driven pain management following tooth extractions. This implementation project has the potential to drive a major improvement in prescribing practices and the management of pain following tooth extractions in the field of dentistry. As new strategies for pain management are ready to be introduced into clinical practice, they can be readily integrated into this platform in a timely and transparent manner [22].

**Figure 1:**
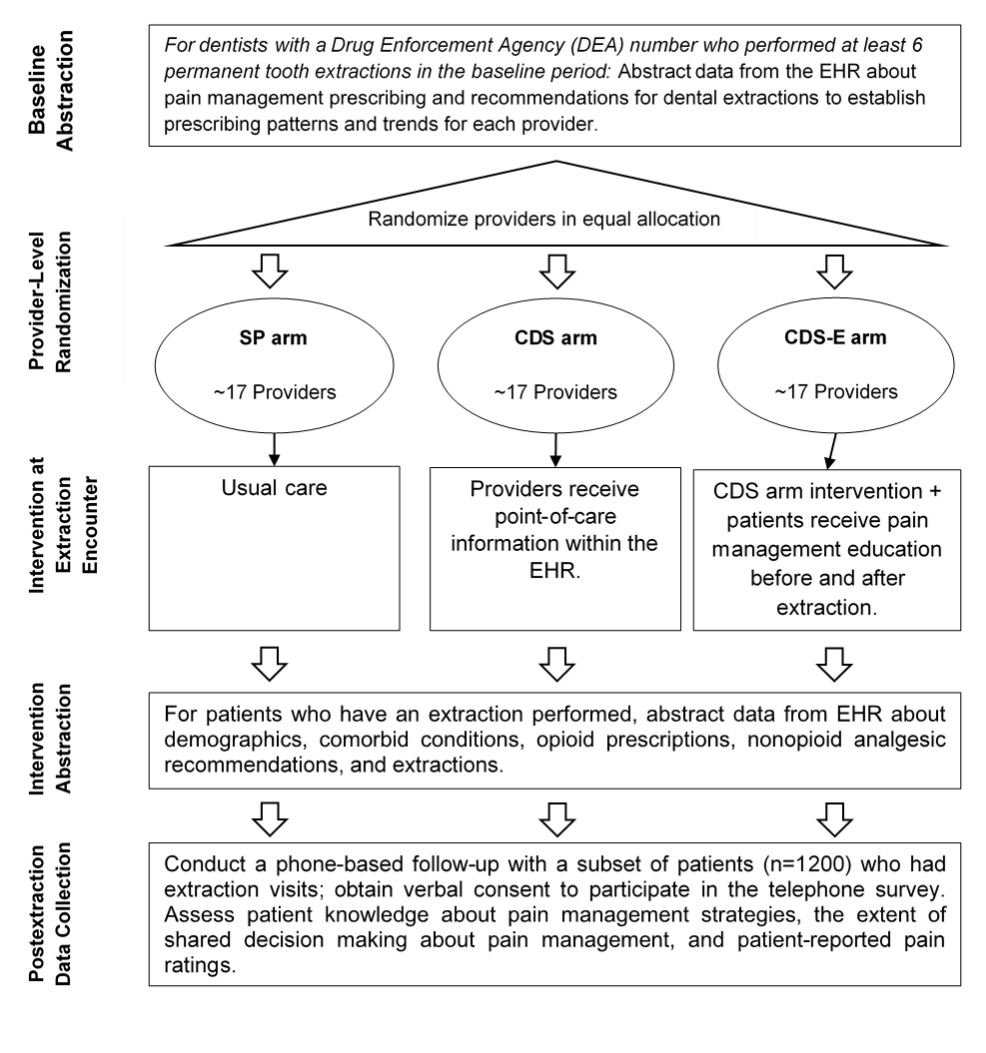
Study overview. CDS: clinical decision support; CDS-E: clinical decision support with patient education; EHR: electronic health record; SP: standard practice.

The primary objective of this project is to test the ability of CDS to support the de-implementation of opioid analgesics and to advance the use of nonopioid (NSAID+APAP) analgesics to manage pain following dental extractions. Using a prospective, 3-arm, cluster randomized trial design with dentists as the unit randomized and patient-level prescribing data as the primary outcome, the study team will compare different strategies to reduce the reliance on opioids and increase the use of alternative pain management approaches utilizing information support tools aimed at both providers and their patients (Figure 1). The primary objective is to test the efficacy of 2 interventions (CDS with and without patient education), compared to treatment as usual to decrease opioid prescribing for dental extractions. Drawing from encounter-level data from the electronic health record (EHR), we will compare study arms on the percentage of extraction encounters with an opioid prescribed.

Secondary objectives are to (1) test the efficacy of 2 interventions (CDS with and without patient education) compared to standard practice to increase exclusive nonopioid pain management for dental extractions, (2) compare the degree to which each of the 3 study arms (CDS with and without patient education and standard practice) facilitates shared provider and patient decision making concerning pain management options for dental extractions, and (3) explore whether the study interventions lead to differences in patient experiences with postextraction pain.

## Methods

### Study Design

This study is a prospective, 3-arm, cluster randomized trial (Phase III) in which up to 60 dentists (accrual goal: n=51) practicing at HealthPartners dental clinics will be randomized in a 1:1:1 allocation ratio to either standard practice (SP) or 1 of 2 CDS arms (CDS or CDS-E). Patients (up to n=8400; accrual goal: n=6900) will be exposed to the study arm to which their dentist was assigned. Opioid prescribing measured at the encounter level will serve as the primary outcome. Additional outcomes will also be measured at the patient level, including a recommendation or prescription for nonopioid analgesics as indicated in the EHR and patient-reported outcomes concerning shared decision making and pain levels as identified through patient surveys.

It was impractical to randomize at the patient level for a study that equips providers with CDS. Instead, this cluster-randomized trial design randomizes dentists to 1 of 3 study arms. This design maximizes the number of randomized units (ie, providers) as compared to clinic-level randomization. This design also solves the practical issue of providers who deliver care across several clinics and different study arms that would occur in clinic-level randomization. The institutional review board at HealthPartners Institute reviewed and approved the protocol for this study.

### Study Site

HealthPartners is the largest consumer-governed, nonprofit health care organization in the country, providing care, coverage, research, and education to improve health and well-being in partnership with its members, patients, and community. The organization includes a multispecialty medical and dental group practice that includes 75 dentists and 24 dental clinics in Minnesota. For the past 18 years, the medical and dental clinics have fully implemented EHRs with integrated diagnosis codes. The HealthPartners Institute utilizes these systems to conduct research.

### Eligibility

Study-eligible providers are practicing dentists including oral surgeons at HealthPartners with a current Drug Enforcement Agency license who performed permanent tooth extractions at a minimum of 6 encounters in the baseline period 1 year prior to implementing the intervention. Study-eligible dental patients are aged 16 years and older and had a permanent tooth extraction performed by a study-eligible HealthPartners provider. Patients are excluded if they have opted out of research at HealthPartners.

### Recruitment

Implementation of the CDS is considered a HealthPartners Dental Group initiative. The project is being implemented with the aim of reducing opioid prescribing. Consent has been provided by dental leadership in accordance with HealthPartners practices. Providers randomized to the CDS or CDS-E group will be exposed to the CDS, which is integrated into the EHR. Patients who receive care from providers randomized to the CDS-E arm will also be exposed to study-specific patient education and receive additional educational resources. Thus, as part of the study design, a new section of the EHR is activated for providers in the CDS and CDS-E arms, whereas these EHR resources are left dormant for providers in the SP arm. For the primary study objective, the study does not consent providers or patients.

For the survey-based secondary study objectives, a subset of patients undergoing tooth extractions will be recruited for a telephone survey after the index dental visit. Trained survey administrators will ask these patients to consent verbally for participation via telephone. Stratified sampling will be used to ensure roughly equal representation of patient sample sizes in each study arm and to ensure that patients linked to each provider are sampled for surveys. Sampling weights will be used in the analysis to re-weight the sample results to resemble the population of patients receiving extractions.

### Measures to Minimize Bias

The randomization of providers to the study arms will utilize stratification on provider type (oral surgeon vs other) and level of provider opioid prescribing during the baseline period.

Providers will be randomly allocated 1:1:1 through a computer-generated program to SP, CDS, or CDS-E. The randomization will be conducted by the study statistician and use a provider identification number with no recognizable meaning, ensuring that the study statistician is blind to provider identity (name). The crosswalk containing the provider identification number, provider name, and study arm will be kept in a secure file and accessible only by the study programmer. In order to minimize contamination across study arms, only providers in the CDS or CDS-E arms will be able to access the CDS. Similarly, patients who receive care from providers in the CDS-E arm will have access to supplemental patient education. As such, both groups of providers will know their assignment. The study team will not disclose the study’s purpose, objectives, or outcomes measures directly to patients until after the encounter or after the participant has been surveyed about their experience, if selected.

Patients will be exposed to the intervention to which their provider has been randomized. At each patient encounter, the CDS web application will collect patient-level data from the EHR (eg, medical conditions, current medications, social history). Providers assigned to the CDS or CDS-E arms will be able to access the information using the CDS tool. Providers assigned to the SP control arm will continue to use their usual methods to assess and address pain management for dental procedures, without any effort from the study to influence their prescribing of analgesics following the dental extraction. They will not be shielded from other outside influences.

At the index encounter (the initial patient visit for a dental extraction where all the study criteria are met), the patient is assigned a unique study identifier that is used to link patient encounter data over time. All index and subsequent encounter data for eligible patients are stored in a limited de-identified analysis dataset. The index encounter is the only patient encounter utilized in the primary analysis. Staff administering the patient survey will not know which arm providers, and thereby survey respondents, have been assigned.

### Study Intervention

**Figure 2:**
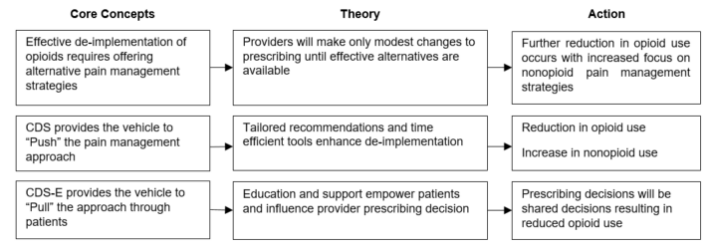
Theoretical framework.

The study intervention follows the theoretical framework outlined in Figure 2.

#### SP

Providers will not receive point-of-care CDS, serving as the control group representing usual care. Patients will receive usual dental care.

#### CDS

Providers will receive point-of-care CDS to guide pain management recommendations and prescribing for patients who receive dental extractions (see Multimedia Appendix 1). The CDS will highlight (1) potential medication interactions between the patient’s current medications and commonly recommended analgesics for patients following dental extractions, (2) relevant health conditions and identified substance use disorders that may impact pain management strategies, and (3) automated access to the state’s Prescription Monitoring Program. The decision support identifies these potential medication interactions and medical conditions for all analgesic classes (APAP, NSAID, opioid). Patients will receive usual dental care.

#### CDS-E

Providers will receive point-of-care CDS to guide pain management recommendations and prescribing related to dental extractions. Providers will receive the same point-of-care CDS as in the CDS arm (see Multimedia Appendix 1). In addition, patients will receive an educational handout by front desk staff when checking in for the extraction procedure (see Multimedia Appendix 2 and Multimedia Appendix 3). The handout compares opioid and nonopioid pain medications with respect to their risks, benefits, and effectiveness for managing pain. This handout intends to initiate a conversation between the patient and provider about patient needs, goals, concerns, and pain management preferences. This handout also includes information about other pain management after a dental extraction. This handout intends to normalize the experience of some discomfort, help to set realistic expectations, and help patients decide whether to contact their provider for additional help to manage their pain after extraction. Patients will receive usual dental care.

### Clinical Decision Support Design

The CDS is designed to bring all available clinical information into one interface, saving time searching for the relevant information that would inform the best analgesic options for that individual patient. The CDS identifies potential drug interactions and side effects related to the medications the patient is taking and the potential analgesic options. The CDS messages provide this information to the dentist so it is considered in the analgesic prescribing decision. The messages were informed by information from Micromedex [23], which is considered a highly reliable and current evidence-based source. Medical conditions also need to be considered. The relevant conditions that should be considered when determining the analgesic to prescribe were identified and reviewed by medical experts in the related specialties to ensure that accurate information was provided. The record is also examined for substance use, a history of a substance use disorder, a history of overdose, and a naloxone prescription. Access to the prescription drug monitoring programs is also contained within the CDS. The messaging in the CDS is brief, with the intent of informing a discussion with the patient about why this analgesic recommendation is personalized to them while considering both safety and effectiveness.

### Patient Education Development

A 9-question survey was completed by 232 patients or plan members through the HealthPartners myVoice panel, providing insights regarding pain expectations and beliefs and preferences for pain management following dental extractions. Findings were used to develop pain management–focused messaging for the enhanced intervention arms of the trial.

### Clinic Workflow Considerations

In order to optimally situate the timing of the CDS in the EHR, study team members conducted 15 observations of dental extractions with providers delivering standard care using a structured checklist during the study preparation phase, with a focus on provider-patient interactions as well as provider use of the EHR as it relates to analgesic prescribing for extractions.

### Fidelity Monitoring

Fidelity is monitored by tracking whether the CDS was opened during the clinical encounter. The CDS link is highlighted in the EHR when a treatment plan exists, reminding the provider to open the CDS. However, a treatment plan may not exist for every extraction, for example if the treatment was not determined prior to the visit. We are able to determine if the CDS was opened by the provider, as well as whether a treatment plan existed. Monitoring these data points informs whether further provider training is needed to improve fidelity.

We also monitor whether the patient education material was printed for all eligible patients. These data inform whether further training of support staff is needed to ensure fidelity.

### Data Sources and Endpoints

A data pull from the EHR at the end of the study will serve as the data source for the primary and secondary objectives (see Table 1). Specifically, prescriptions for opioids medications and nonopioid analgesic medications will be available from the EHR. This includes recommendations by the provider to use over-the-counter nonopioid analgesic medications. The EHR will also provide information on prescription quantities, patient demographics, and the dentist conducting the extraction.

**Table 1:** Objectives, endpoints, and data sources.

Objectives		Endpoints	Data source
**Primary**			
	To test the efficacy of 2 interventions (clinical decision support with and without patient education), compared to treatment as usual to decrease opioid prescribing for dental extractions	Differential pre- to postintervention change by study arm in the percentage of extraction encounters with an opioid prescribed on the day of the extraction encounter	EHR^a^
**Secondary**			
	To test the efficacy of 2 interventions (clinical decision support with and without patient education), compared to treatment as usual to increase exclusive nonopioid pain management for dental extractions	Differential pre- to postintervention change by study arm in the percentage of extraction encounters at which a provider prescribed or recommended nonopioid analgesics (ibuprofen, naproxen, aspirin, or acetaminophen) and did not prescribe opioids on the day of the extraction encounter	EHR
	To compare the degree to which each of the 3 study arms (clinical decision support with and without patient education and treatment as usual), facilitates shared provider and patient decision making concerning pain management options for dental extractions	Study arm comparison of the mean of the patient-reported shared decision-making composite score (composite of 3 components concerning management of postextraction pain options: effort to explain, listen, and personalize), 3-6 days after the extraction encounter	Patient survey 3-6 days following the extraction encounter
	To explore whether the study interventions lead to differences in patient experiences of postextraction pain	Study arm comparison of the average patient-reported pain following the extraction, 3-6 days after the extraction encounter	Patient survey 3-6 days following the extraction encounter

^a^EHR: electronic health record.

A survey conducted within 3-6 days of the extraction encounter in a sample of patients with extractions will be the data source for the remaining 2 secondary objectives concerning patient reports of shared decision making and pain. The survey contains 17 items and is designed to be completed over the phone in less than 5 minutes. Survey constructs include overall satisfaction with the visit, shared decision making, confidence in ability to management postextraction pain, pain experienced in the days following extraction, and strategies used to manage pain. Questions are based on items with known psychometric properties where available, such as CollaboRATE for shared decision making [24] and the numerical pain rating scale. Demographic questions are based on standard items from Federal Surveillance Surveys such as the Behavioral Risk Factor Surveillance Survey. Where concepts were sought without existing available questions or scales, survey questions were written using best practices for survey questions in order to minimize measurement error and respondent burden [25].

Patient survey data will be collected via phone survey by the research organization’s Center for Evaluation and Survey Research and managed using research electronic data capture (REDCap). REDCap is a secure, web-based application designed to support data capture for research studies, providing (1) an intuitive interface for validated data entry, (2) audit trails for tracking data manipulation and export procedures, (3) automated export procedures for seamless data downloads to common statistical packages, and (4) procedures for importing data from external sources [26].

### Safety Assessments

The study does not include objectives or endpoints concerning safety. However, the monitoring activities will be conducted to assess possible harm.

### Sample Size and Power

EHR data from 2018 indicate there were 6900 unique patients aged 16 years and older with a permanent tooth extracted by 51 dentists in the baseline period and also an estimated 6900 unique patients aged 16 years and older with a permanent tooth extracted by 51 dentists during the intervention period. The first visit for a patient in a specific time period (baseline, intervention) at which a patient is eligible for the study serves as the index encounter for the patient to be utilized for the analysis. The study analysis is expected to have at least this many patients. Fully 40% of encounters in the baseline period with patients having a permanent tooth extraction included a prescription for opioid use, and the provider-level opioid use intraclass correlation was 0.3. Assuming the use of a generalized linear mixed model, with an alpha of .05 and 2-sided tests, the planned analysis for the primary objective can detect a differential raw reduction of 23% from pre- to postintervention opioid prescribing when comparing CDS or CDS-E patients (40% with opioid prescriptions pre-implementation to 15% postimplementation) to SP patients (40% with opioid prescriptions pre-implementation to 38% postimplementation) with 80% power.

### Statistical Analysis Plan

Baseline characteristics of study participants will be summarized with mean and standard deviation for interval data and proportions for categorical data. General and generalized linear mixed models will be used to test study arm differences in endpoints. Models will include terms for study arm, time (for models assessing change from pre- to postintervention), and their interaction. Covariates to be included in models will be described in the Manual of Procedures. A random intercept for provider will be included in models to account for the cluster-randomized design with providers as the unit randomized. Differential change will be compared for each study arm via a series of planned contrasts. Study arm contrasts will be tested at an alpha of .05, and all tests will be 2-sided. Model-predicted means and proportions along with 95% confidence intervals will be used to assess the magnitude, direction, and precision of intervention effects.

## Results

The study was implemented in February 2020. Initial monitoring indicates that activation of the CDS is very high when an alert is present. The alert is activated by a treatment plan for an extraction. We know that a treatment plan may not exist for every visit because the provider and patient may not make the decision to extract the tooth until the day the tooth is actually removed. As providers become more familiar with the CDS and start to make small changes in their workflow within the EHR, the potential exists for higher levels of utilization.

One month after implementing the study in the clinics, enrollment was halted due to the COVID-19 pandemic. As public health strategies were implemented to reduce the transmission of the virus, federal, state, and professional organizations recommended that dental clinics postpone any nonemergent or elective dental care. In mid-March, HealthPartners implemented these recommendations, and they continue to be in place at the time of this submission. Once dental practice is able to resume, questions remain regarding what changes in operations and equipment will be needed to prevent the spread of COVID-19. We anticipate these changes will have some impact on patient flow and subsequent study accrual rates.

We continue to see changes in opioid prescribing patterns over time. HealthPartners has taken actions to reduce prescribing. These include reducing the default number of opioid tablets that can be prescribed and the creation of opioid guidelines. The Minnesota Legislature and insurance plans have taken additional actions that provide additional regulatory oversight and monitoring of provider prescribing of opioids. These actions are all good if they lead to appropriate reductions in opioid prescribing but may limit our ability to test our intervention strategies, as these strategies are being used within a broader institutional, state, and national context of increased attention on reducing opioid prescriptions.

## Discussion

Overreliance on opioids across the health care system contributes to the ongoing opioid use disorder and overdose crisis in the United States. Opioids continue to be used in dentistry following tooth extractions. While opioids are clinically appropriate in some circumstances, evidence shows that, in many cases, nonopioid analgesics are as effective and safer for dental extractions. Strategies are needed that can help the field of dentistry to de-implement the routine use of opioids for dental extractions. CDS tools have demonstrated efficacy at improving various health outcomes in medicine and are showing similar potential in dentistry [27]. A CDS that saves time by bringing all relevant patient information and current evidence to the provider in one interface has the potential to improve clinical decision making as well as patient outcomes. This trial examines both the potential for CDS to improve dentists’ prescribing and the potential additive effect of providing patients with relevant analgesic safety and efficacy information prior to the dental appointment when prescribing decisions are made. The study could highlight effective strategies for de-implementing opioids, improving patient care, and protecting public health. We plan to disseminate the results in relevant journals and through channels made available through the HEAL Initiative.

## Appendix

Multimedia Appendix 1 Clinical decision support.

Multimedia Appendix 2 Creating a plan to manage pain.

Multimedia Appendix 3 Caring for yourself after having a tooth removed.

Multimedia Appendix 4 CONSORT-eHEALTH checklist (V 1.6.1).

## Abbreviations

APAP: acetyl-para-aminopheno (aka paracetamol or acetaminophen)
CDS: clinical decision support
CDS-E: clinical decision support with patient education
EHR: electronic health record
NSAID: nonsteroidal anti-inflammatory drug
REDCap: Research Electronic Data Capture
SP: standard practice
