# Supplementary figures and images for: De-Implementing Opioid Use and Implementing Optimal Pain Management Following Dental Extractions (DIODE): Protocol for a Cluster Randomized Trial
Source: JMIR Res Protoc. 2021 Apr 12;10(4):e24342. doi: 10.2196/24342 (PMC8076983; doi:10.2196/24342)

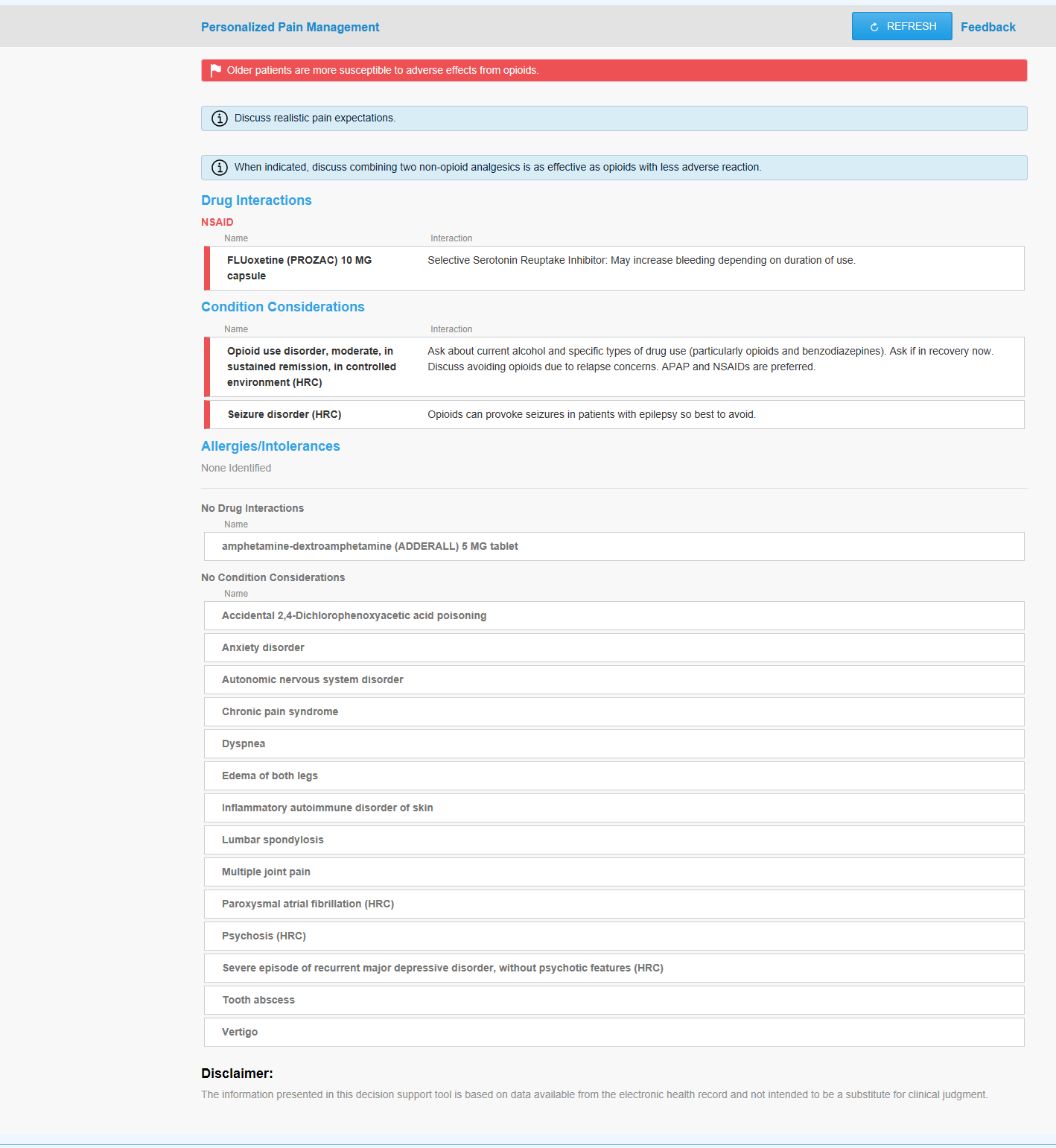

Supplement: Multimedia Appendix 1 [file resprot_v10i4e24342_app1.png]
