# Supplementary material for: De-Implementing Opioid Use and Implementing Optimal Pain Management Following Dental Extractions (DIODE): Protocol for a Cluster Randomized Trial
Source: JMIR Res Protoc. 2021 Apr 12;10(4):e24342. doi: 10.2196/24342 (PMC8076983; doi:10.2196/24342)
Supplement: Multimedia Appendix 2 [file resprot_v10i4e24342_app2.pdf]

Creating a Plan to Manage Pain

Pain is normal after having a tooth removed, and your dentist cares about your comfort. We want to help you manage your pain so that you can get back to doing what's important to you.

Pain medication

During the first few days after surgery, pain medication can help manage your pain. Taking ibuprofen (Advil) and acetaminophen (Tylenol) is often the best way to manage pain with the fewest side effects. Follow your dentist's instructions for taking any type of pain medication.

Medications	Benefits	Risks and Side Effects
**Non-opioid pain medications**<br>• Ibuprofen<br>• Acetaminophen<br>• Naproxen (Aleve)	• Not habit forming<br>• Few side effects with short-term use<br>• Does not require a prescription	• May not be advised for some people with certain medical conditions<br>• May increase bleeding for some individuals
**Opioids**<br>• Codeine<br>• Oxycodone (OxyContin, Percocet)<br>• Hydrocodone (Vicodin)	• Relieves severe short-term pain	• Nausea<br>• Drowsiness<br>• Vomiting<br>• Constipation<br>• Slowed breathing<br>• May make pain worse if used for extended periods<br>• May lead to addiction<br>• May be harmful or fatal if too much is taken at once (overdose)<br>• May be harmful or fatal when taken with other medications, supplements, or alcohol and other drugs<br>• Unused medication could be misused by others

Talk to your dentist

Your comfort and safety are important for your recovery. To help manage your pain, tell your dentist:

- All of your health conditions—even those that don't seem related to your dental care
- All medications, vitamins and supplements you're taking—even those without a prescription
- If you are pregnant or breastfeeding

Together, you and your dentist can create a plan to safely manage your pain in a way that works best for you.

(7/2019) ©2019 HealthPartners
