# Supplementary material for: De-Implementing Opioid Use and Implementing Optimal Pain Management Following Dental Extractions (DIODE): Protocol for a Cluster Randomized Trial
Source: JMIR Res Protoc. 2021 Apr 12;10(4):e24342. doi: 10.2196/24342 (PMC8076983; doi:10.2196/24342)
Supplement: Multimedia Appendix 3 [file resprot_v10i4e24342_app3.pdf]

Caring for Yourself after Having a Tooth Removed

A recovery plan can help you heal after oral surgery. Follow your dentist's instructions and create a plan together to help manage your pain.

Managing care the first day after surgery

To reduce the chance of bleeding and swelling during your first day after surgery:

- Do not smoke.
- Do not drink through a straw.
- Start by eating soft food only.
- Be gentle when rinsing your mouth.
- Apply a cold pack to the area of your face near where your tooth was removed. This can reduce swelling—the cause for most pain after oral surgery.

Managing pain the first week after surgery

It is normal to have some pain for up to 7 days after having a tooth removed. Knowing when the pain is too much is important. Use this scale to help you rate your pain, with 0 being no pain and 10 being the worst pain you've ever had.

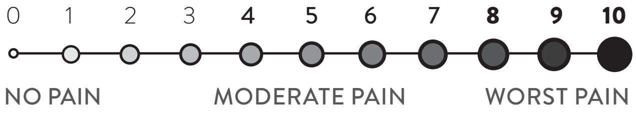

- **If your pain is 1 to 3:** Your pain is well managed. Continue to follow your dentist's instructions.
- **If your pain is 4 to 6:** Your pain could be lessened by doing the following.
  - » Make sure you are taking your medication as directed.
  - » Make sure you are following your dentist's instructions.
  - » Contact your dentist if you have followed directions and your pain does not get better.
- **If your pain is 7 or higher:**
  - » If you have been following your dentist's instructions, including taking medications, contact your dentist.
  - » If you just started following the instructions, wait 24 hours and rate your pain again. If your pain is still 7 or higher, contact your dentist.

What can affect your pain

- **Brushing.** Keeping your mouth clean helps reduce your chance of infection. Brush and floss gently but avoid the surgical site. Soreness and swelling may keep you from brushing all areas. Clean your mouth the best that you can.
- **Swelling.** If you still have swelling the day after your surgery, apply a warm, moist compress to the area of your face near where the tooth was removed (10 minutes on, 10 minutes off).
- **Fever.** A slight fever up to 100.5°F (38.6°C) is common during the 48 hours after surgery.
- **Dry socket.** After your tooth is removed, a clot will form in the area. If this clot comes off before the area heals, this is called a dry socket. This may happen 3 to 5 days after the tooth is removed and can cause increased pain in the area.

CONTINUED

How to manage your pain

- **Pain medication.** During the first few days after surgery, pain medication can help relieve your pain. As your pain gets better, you can reduce or stop pain medication. Some medications (such as opioids) are meant to be used for a short time only and often have side effects. Safely dispose of all unused opioids at a local collection site. Follow your dentist's instructions for taking any type of pain medication.
- **Deep breathing.** When you're in pain, you may find yourself holding your breath or breathing shallowly. This reduces oxygen in your body and strains muscles in and around your head and neck. Deep breathing helps slow your breathing and relax your body and mind. Try deep breathing exercises when your pain is intense.
- **Visual imagery.** This skill uses your thoughts, senses and mental pictures to cause positive changes in your body. Visual imagery can help with the mental and emotional aspects of pain. Do visual imagery exercises several times a day to help manage and reduce pain.
- **Other methods.** Your brain produces chemicals that are natural painkillers. Tap into those painkillers by using aromatherapy, music therapy and other methods to help manage your pain.

When should I contact my dentist?

Call your dental clinic if you have questions, concerns or you experience any of the following:

- Increased swelling after 48 hours
- Fever of 101°F (38°C) or higher or a fever that lasts longer than 48 hours
- Dry socket

After-hours assistance is available by calling HealthPartners CareLine℠ at **612-339-3663**, evenings and weekends.

(7/2019) ©2019 HealthPartners
